# Conservation of the Hydrogen-Bonded Pyridone Homosynthon in Halogen-Bonded Cocrystals

**DOI:** 10.1021/acs.cgd.1c01424

**Published:** 2022-01-10

**Authors:** Nikola Bedeković, Luka Fotović, Vladimir Stilinović, Dominik Cinčić

**Affiliations:** University of Zagreb, Faculty of Science, Department of Chemistry, Horvatovac 102a 10000 Zagreb, Croatia

## Abstract

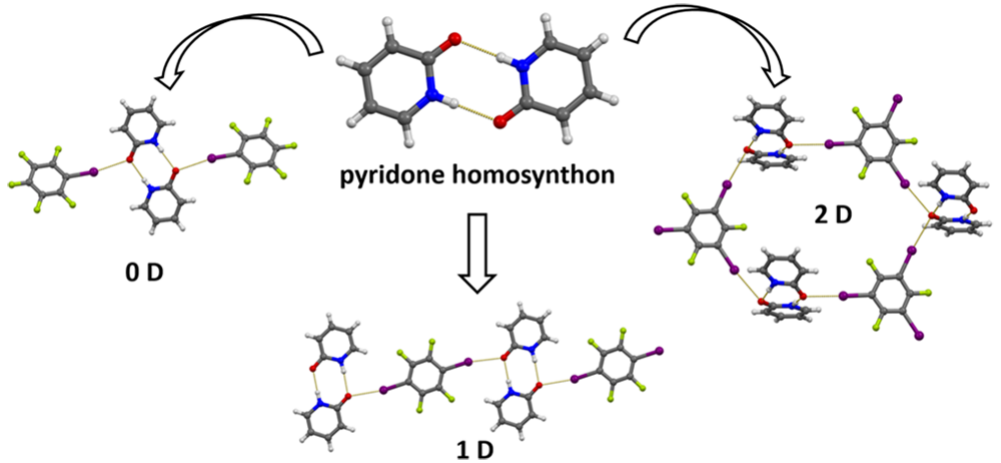

Seven cocrystals of pyridone and perfluorinated halocarbons have been prepared. In all cases pairs of pyridone molecules are connected into dimers by two N–H···O hydrogen bonds, forming the characteristic pyridone homosynthon of R_2_^2^(8) topology. These dimers further act as acceptors of halogen bonds through the two pyridone oxygen atoms, forming two (in six cases) or three (in one case) halogen bonds with the donor molecules. The stoichiometry of the cocrystals obtained and the overall topology of the supramolecular architecture depend primarily on the topicity of the halogen bond donor, with the monotopic donor yielding a cocrystal of 1:1 stoichiometry comprising discrete supramolecular complexes, the ditopic donors cocrystals of 1:2 stoichiometry comprising chains, and the tritopic donor a cocrystal of 1:2 stoichiometry comprising hydrogen- and halogen-bonded layers. The results indicate that the pyridone homosynthon is a robust and reliable supramolecular synthon that is conserved in halogen-bonded cocrystals of pyridone.

## Abstract 2

The hydrogen-bonded pyridone homosynthon is preserved in halogen-bonded cocrystals with perfluorinated halogen bond donors.

## Introduction

The controlled molecular recognition and targeted formation of desired supramolecular moieties using directional intermolecular interactions have always been the main focus of crystal engineering.^1−5^ The success of such an approach largely depends on both the types of the molecules or ions used and their complementarity for binding via a particular type of interaction. In this context, the term supramolecular synthon has been defined to denote a robust connector that can be used for linking molecules in crystal structure in the desired supramolecular motifs, which relies on the complementarity of two functional groups^6−10^ (for example, a carboxylic acid–pyridine heterosynthon^11−17^ or a cyclical carboxylic acid homosynthon^18−20^).

**Scheme 1:**
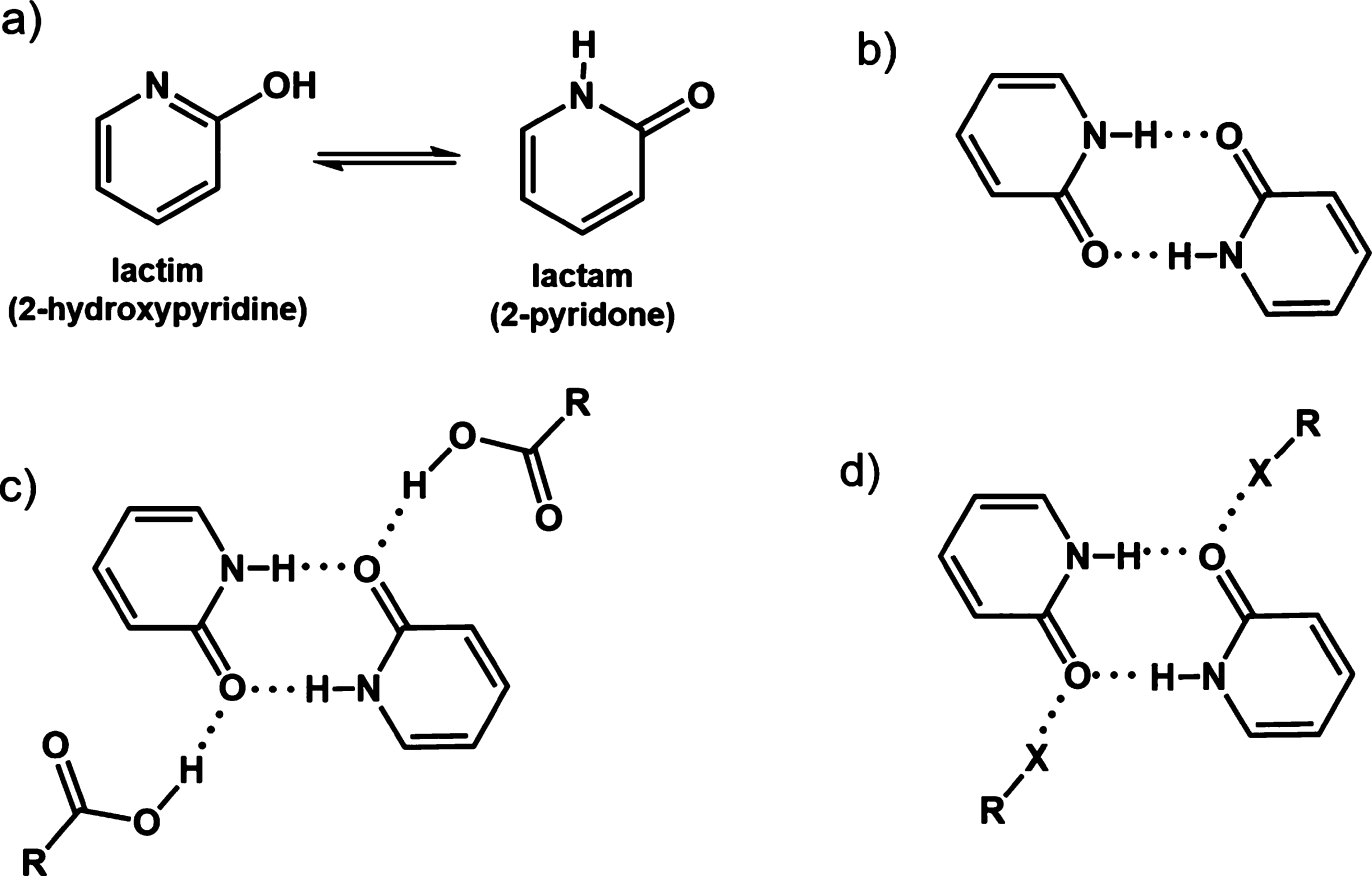
(a) Lactim–Lactam Tautomerization, (b) Cyclic R_2_^2^(8) Pyridone Homosynthon, (c) Hydrogen Bonding of Bis(pyridone) Dimers with Carboxylic Acids, and (d, This Work) Bis(pyridone) Dimers as Halogen Bond Acceptors

Chemical species containing a 2-hydroxypyridine fragment in the solid state preferentially exist as lactam (2-pyridone) tautomers (Scheme 1a) that in multicomponent crystals are prone to aggregate in robust hydrogen-bonded dimers involving two N–H···O hydrogen bonds—the pyridone homosynthon of R_2_^2^(8) topology (i.e., a ring comprising two hydrogen bond donors and two acceptors which includes a total of eight atoms; Scheme 1b).^21,22^ The robustness of such hydrogen-bonded dimers has been well studied in multicomponent solids, including 2-pyridone (**pdon**) derivatives and carboxylic diacids (CAs), in which the acid molecules act as linkers between **pdon** dimers forming chains through O_CA_–H···O_pdon_ hydrogen bonds—the pyridone carbonyl oxygen atom thus being the acceptor of two hydrogen bonds, one with a pyridone molecule and the other with the carboxyl group of the acid (Scheme 1c).^23,24^

The carbonyl oxygen has also been demonstrated to be a viable halogen bond (XB) acceptor, albeit weaker and less reliable than sp^2^ nitrogen.^25−33^ The question therefore arises whether a combination of pyridone and appropriate halogen bond donors would lead to structures in which the pyridone homosynthon is retained (Scheme 1d) or the introduction of a halogen bond^34^ donor would disrupt the pyridone homosynthon, possibly even stabilizing the hydroxypyridine tautomer via an X···N(sp^2^) halogen bond.

**Scheme 2:**
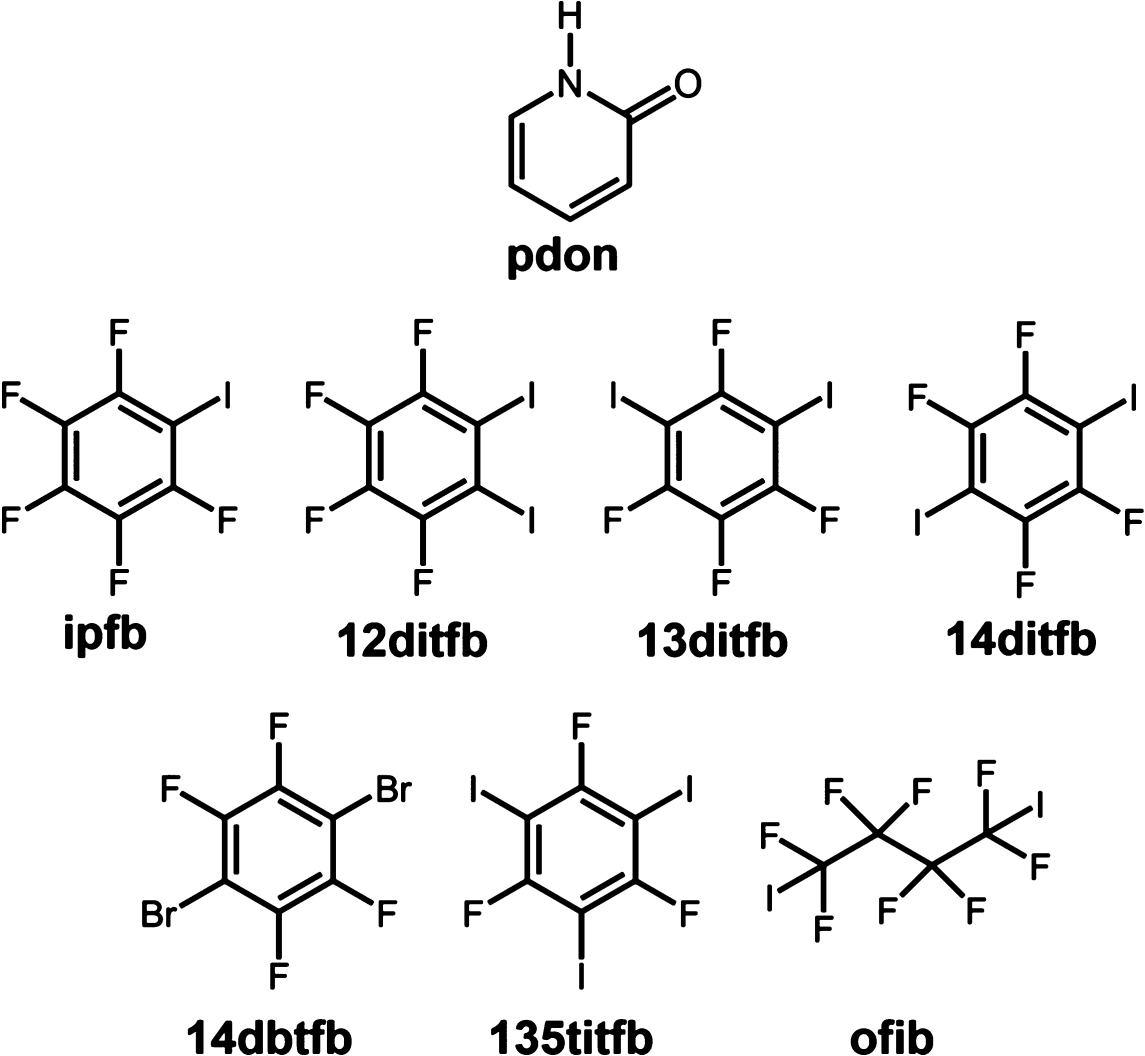
Molecular Representations of Halogen Bond Donors and Acceptors Used

In order to investigate the conservation of the pyridone homosynthon in the presence of halogen bond donors, we have attempted to prepare cocrystals of 2-pyridone (**pdon**) with seven XB donors: iodopentafluorobenzene (**ipfb**), 1,2-diiodotetrafluorobenzene (**12ditfb),** 1,3-diiodotetrafluorobenzene (**13ditfb**), 1,4-diiodotetrafluorobenzene (**14ditfb**), 1,4-dibromotetrafluorobenzene (**14dbtfb**), 1,4-diiodooctafluorobenzene (**ofib**), and 1,3,5-triiodo-2,4,6-trifluorobenzene (**135titfb**) (Scheme 2). The XB donors were selected to include different donor atoms (Br in **14dbtfb** and I in the others) and different numbers of halogen bond donor sites (monotopic **ipfb**, ditopic **12ditfb**, **13ditfb**, **14ditfb**, **14dbtfb**, and **ofib**, and tritopic **135titfb**), as well as, in the case of polytopic donors, different arrangements of XB donor sites and flexibilities of donor molecules (rigid linear **14ditfb** and **14dbtfb**, rigid bent **12ditfb**, **13ditfb**, and **135titfb**, and flexible **ofib**). The prepared compounds were structurally characterized by SCXRD and ART-IR, while their phase purity was investigated by XRPD and thermal analysis.

## Results and Discussion

An overview of the obtained cocrystals is given in Table 1. All seven obtained cocrystals feature hydrogen-bonded dimers (**pdon**)_2_ interconnected via the R_2_^2^(8) pyridone homosynthon. These dimers generally act as ditopic halogen bond acceptors, utilizing the carbonyl oxygen atoms to form a pair of X···O halogen bonds with two neighboring halogen donor molecules. The only exception found was in the case of (**135titfb**)(**pdon**)_2_, where one oxygen atom participates in two halogen bonds (see below).

**Table 1:** Obtained Cocrystals with Corresponding Hydrogen (*d*(N···O), *ϑ*(N–H···O)) and Halogen Bond Geometries (*d*(X···O), *ϑ*(C–X···O)), as Well as the Angles between the Halogen and Hydrogen Bonds Formed with the Same Acceptor (*ϑ*(X···O···N))

	HB		XB		
	*d*(N···O) (Å)	*ϑ*(NHO) (deg)	*d*(X···O) (Å)	*ϑ*(CXO) (deg)	HB/XB *ϑ*(XON) (deg)
(**ipfb**)(**pdon**)	N1–H1n···O1		C1–I1···O1		I1···O1···N1
	2.72(2)	175(2)	2.81(1)	170.4(3)	107.7(6)
(**12ditfb**)(**pdon**)_2_	N1–H1n···O1		C1–I1···O1		I1···O1···N1
	2.770(3)	179.7(1)	3.041(2)	159.29(8)	93.07(8)
(**13ditfb**)(**pdon**)_2_	N1–H1n···O1		C1–I1···O1		I1···O1···N1
	2.769(7)	171.8(2)	2.765(5)	169.9(2)	96.2(2)
	N2–H2n···O2		C2–I2···O2		I2···O2···N2
	2.736(9)	175.7(7)	2.817(5)	176.9(2)	106.5(7)
(**14ditfb**)(**pdon**)_2_	N2–H2n···O1		C1–I1···O1		I1···O1···N2
	2.780(5)	176.4(3)	2.795(4)	168.9(1)	95.1(1)
	N1–H1n···O2		C4–I2···O2		I2···O2···N1
	2.846(5)	173.9(1)	2.807(4)	170.8(1)	92.0(1)
(**14dbtfb**)(**pdon**)_2_	N2–H2n···O1		C1–Br1···O1		Br1···O1···N2
	2.770(5)	173.0(1)	2.876(4)	169.1(2)	91.4(1)
	N1–H1n···O2		C4–Br2···O2		Br2···O2···N1
	2.832(5)	176.1(3)	2.925(4)	172.1(2)	90.2(1)
(**ofib**)(**pdon**)_2_	N2–H2n···O1		C1–I1···O1		I1···O1···N2
	2.774(3)	172.9(1)	2.789(2)	175.60(9)	101.79(8)
	N1–H1n···O2		C4–I2···O2		I2···O2···N1
	2.767(3)	171.9(2)	2.776(2)	172.22(9)	102.24(8)
(**135titfb**)(**pdon**)_2_	N1–H1n···O2		C5–I3···O2		I3···O2···N1
	2.721(5)	177.2(2)	2.840(4)	169.8(2)	99.4(1)
	N2–H2n···O1		C1–I1···O1		I1···O1···N2
	2.844(5)	161.5(3)	2.920(5)	175.7(2)	109.7(2)
			C3–I2···O1		I2···O1···N2
			2.941(4)	172.5(2)	90.1(1)

**Figure 1:**
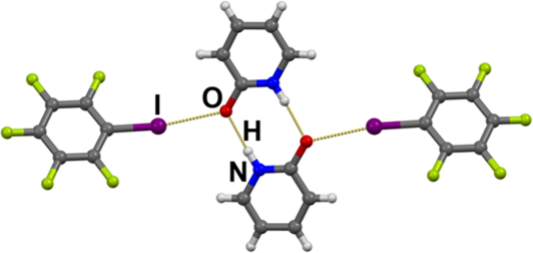
Discrete molecular complex in the crystal structure of (**ipfb**)(**pdon**).

In all the structures where (**pdon**)_2_ dimers act as ditopic XB acceptors, the topology of the supramolecular architecture which is achieved in the cocrystal will depend on the topicity of the XB donor used. The monotopic XB donor **ipfb** participates in the binding via an I···O halogen bond, leading to a cocrystal of overall 1:1 stoichiometry. It comprises discrete molecular complexes, in which two **ipfb** molecules bind to a centrosymmetric hydrogen bonded (**pdon**)_2_ dimer (Figure 1). The angle between the halogen and the hydrogen bond of 107.7° (see Table 1) is one of the largest among the studied complexes—as there are no strong intermolecular interactions interconnecting the (**pdon**)_2_(**ipfb**)_2_ complexes, the two donors interacting with the same acceptor are free to arrange themselves in a sterically more favorable manner.

**Figure 2:**
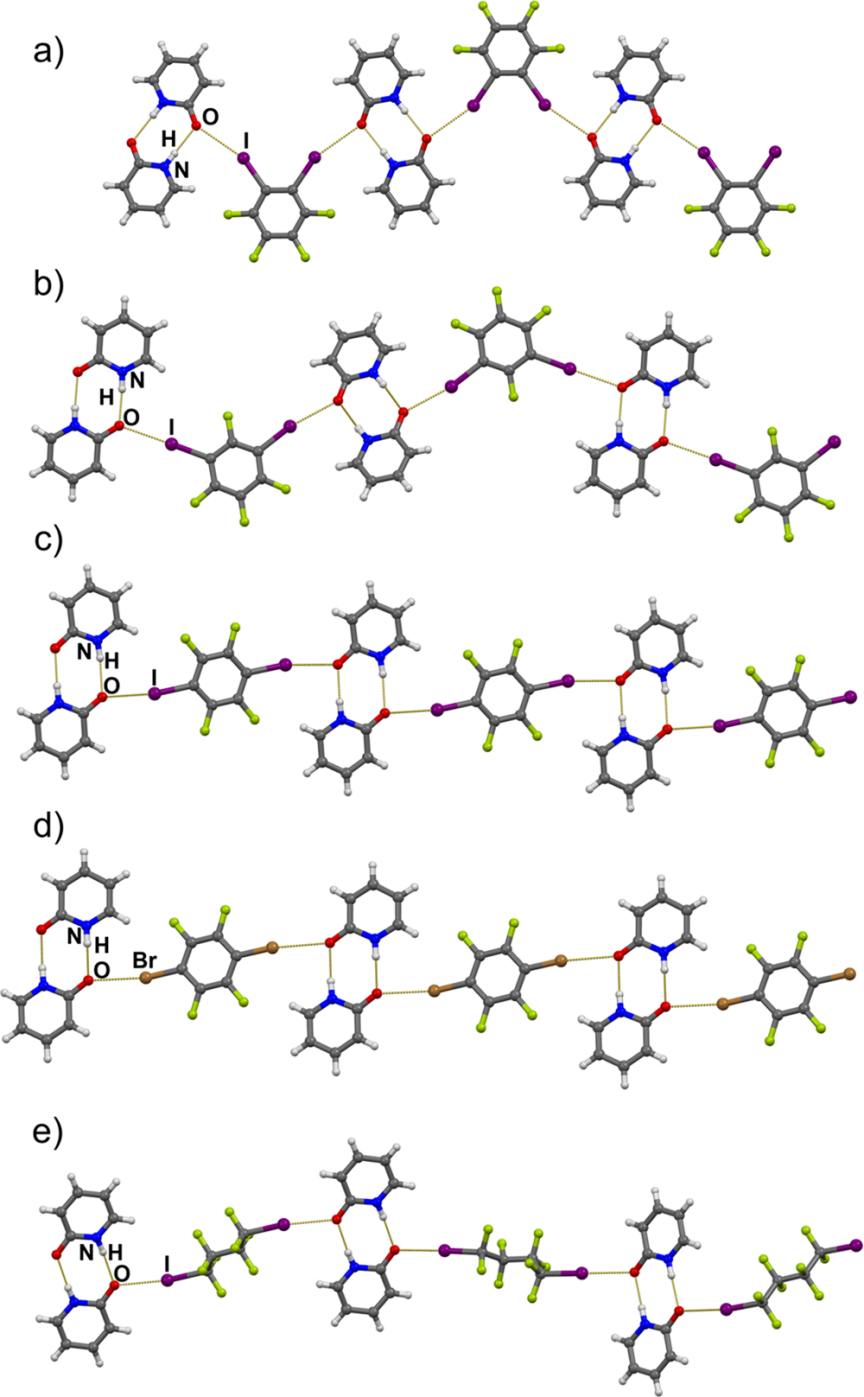
Halogen- and hydrogen-bonded chains in crystal structures of (a) (**12ditfb**)(**pdon**)_2_, (b) (**13ditfb**)(**pdon**)_2_, (c) (**14ditfb**)(**pdon**)_2_, (d) (**14dbtfb**)(**pdon**)_2_, and (e) (**ofib**)(**pdon**)_2_.

When ditopic XB donors are used, cocrystals of 1:2 stoichiometry, (**12ditfb**)(**pdon**)_2_, (**13ditfb**)(**pdon**)_2_, (**14ditfb**)(**pdon**)_2_, (**14dbtfb**)(**pdon**)_2_, and (**ofib**)(**pdon**)_2_, are obtained. Here each XB donor forms two halogen bonds toward the carbonyl oxygen atoms of two (**pdon**)_2_ dimers, giving rise to chains (Figure 2).

All of the halogen bonds are relatively short, with X···O contacts between 13.1% (in (**12ditfb**)(**pdon**)_2_) and 21.0% (in (**13ditfb**)(**pdon**)_2_) shorter than the sum of the corresponding van der Waals radii (Table S2 in the Supporting Information). In (**12ditfb**)(**pdon**)_2_ both the XB donor molecule and the (**pdon**)_2_ dimer are positioned on special positions of the space group, making all halogen bonds (as well as hydrogen bonds) symmetrically equivalent. In (**13ditfb**)(**pdon**)_2_ however, only the (**pdon**)_2_ dimers are positioned on crystallographic inversion centers, with the **13ditfb** molecule in a general position, forming two inequivalent halogen bonds with two (**pdon**)_2_ dimers that are independent by symmetry. The hydrogen bonds in the two dimers differ significantly in their lengths and angles, with the shorter and more linear hydrogen bond corresponding to the dimer that forms longer halogen bonds with the **13ditfb** molecule. Cocrystals of the two geometrically equivalent XB donors **14ditfb** and **14dbtfb** are isostructural, but as is expected, there are considerable differences in Br···O and I···O halogen bond lengths (*rs*(Br···O) = 13.2% and 14.7%; *rs*(I···O) = 19.8% and 20.1%), which can be attributed to the poorer XB donor abilities of the bromine atom in comparison to the iodine atom. This difference in halogen bond lengths is also reflected in the thermal stabilities and TG decomposition profiles of these two cocrystals: decomposition of (**14ditfb**)(**pdon**)_2_ is a single-step process starting at 107 °C, while decomposition of (**14dbtfb**)(**pdon**)_2_ proceeds in two steps, the first of which (from ca. 60 °C to ca. 140 °C) is characterized by a reduction in mass by 63.2%, which corresponds to the loss of **14dbtfb** (*w*_calc_ = 62%), although it does occur considerably below its boiling point (*t*_bp_ = 157 °C).^35^ In (**14ditfb**)(**pdon**)_2_ and (**14dbtfb**)(**pdon**)_2_ the (**pdon**)_2_ dimers are not placed on crystallographic inversion centers, and the two hydrogen bonds interconnecting the **pdon** molecules differ slightly in length and angle. Unlike the case in (**13ditfb**)(**pdon**)_2_, however, in both cases the oxygen atom participating in the shorter hydrogen bond also participates in the shorter halogen bond. This is somewhat unexpected, as a pair of Lewis acids interacting with the same base ought to exhibit anticooperativity. Indeed, the anticooperativity of the two interactions seems to be confirmed by a comparison of the corresponding hydrogen bond lengths in (**14ditfb**)(**pdon**)_2_ and (**14dbtfb**)(**pdon**)_2_—the hydrogen bonds in (**14dbtfb**)(**pdon**)_2_ (involving the weaker XB donor and longer halogen bonds) are shorter than those in (**14ditfb**)(**pdon**)_2_. It is therefore likely that the differences in the lengths of inequivalent hydrogen (and halogen) bonds observed in these compounds are due to overall crystal packing. In the structure of (**ofib**)(**pdon**)_2_ the (**pdon**)_2_ dimers are also approximately centrosymmetric; however, this pseudosymmetry does not affect the neighboring molecules, as the two **ofib** molecules which form halogen bonds with the dimer have different conformations: one (positioned on an inversion center) in an extended all-*trans* conformation (torsion angle involving the four carbon atoms of 180°) and the other (on a 2-fold axis) in a bent *gauche* conformation (torsion angle of 55.5°). Interestingly, the (**pdon**)_2_ dimers themselves are more closely centrosymmetric than was the case in (**14ditfb**)(**pdon**)_2_ and (**14dbtfb**)(**pdon**)_2_, with both hydrogen bonds within the dimer being almost identical (differing in length by less than 0.01 Å and in angle by only ca. 1°). It appears therefore that the conformational flexibility of the XB donor has in this case allowed for optimization of the crystal packing, avoiding the necessity of deforming the hydrogen bonding within the pyridone homosynthon.

**Figure 3:**
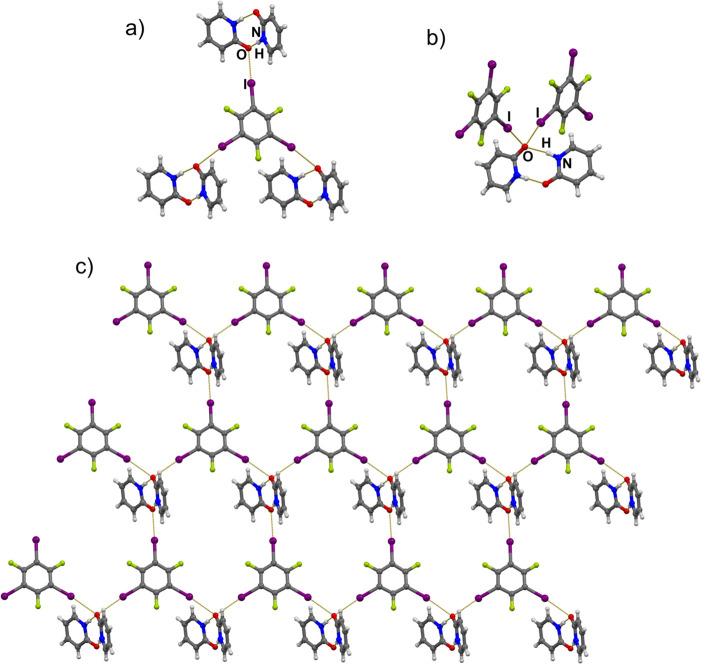
(a) Three X···O halogen bonds formed in the crystal structure of (**135titfb**)(**pdon**)_2_. (b) Two halogen bonds and a hydrogen bond formed by the pyridone oxygen as a tritopic acceptor. (c) 2D network of **135titfb** and (**pdon**)_2_ units.

The (**pdon**)_2_ dimers are present also in the cocrystal with the tritopic donor, **135titfb**, which forms three short I···O halogen bonds with carbonyl oxygen atoms (Figure 3a). However, this does not lead to a cocrystal of the expected 2:3 stoichiometry but rather a (**135titfb**)(**pdon**)_2_ cocrystal, in which the (**pdon**)_2_ dimer acts as an acceptor of three halogen bonds. This is achieved by one oxygen atom in the dimer functioning as a tritopic acceptor of one hydrogen and two halogen bonds (Figure 3b). This results in layers with each (**pdon**)_2_ dimer bridging between three **135titfb** molecules and vice versa (Figure 3c). The overall geometry about the oxygen atom is similar to that found in halogen-bonded *N*-oxides where the oxygen can be an acceptor of three halogen bonds.^36^ The presence of two halogen bonds on the same oxygen has a considerable effect on the hydrogen bond geometry, making it one of the longest and the least linear in the series. In order to accommodate the second halogen bond, the geometry of the (**pdon**)_2_ dimer is also deformed, unlike the case in all of the remaining structures where the two pyridone rings are coplanar, here they are at an angle of ca. 30°. All of this allows for an approximately tetrahedral coordination of the oxygen atom.

All of the above observations lead to the conclusion that the hydrogen-bonded (**pdon**)_2_ motif is a robust and reliable supramolecular synthon in multicomponent halogen-bonded solids. The observed correlation between the cocrystal stoichiometry and XB donor topicity, together with the rigidness of the (**pdon**)_2_ fragment, makes such systems potentially utilizable in practice in the design and synthesis of the desired halogen-bonded structures.

## Supporting Information Available

ORTEP representations of formula units, PXRD patterns, DSC and TG curves, IR spectra, crystallographic data, halogen bond lengths and their relative shortenings, synthetic details, and X-ray diffraction and thermal analysis details (PDF)

## Accession Codes

CCDC 2125558–2125564 contain the supplementary crystallographic data for this paper. These data can be obtained free of charge via www.ccdc.cam.ac.uk/data_request/cif, or by emailing data_request@ccdc.cam.ac.uk, or by contacting The Cambridge Crystallographic Data Centre, 12 Union Road, Cambridge CB2 1EZ, UK; fax: +44 1223 336033.
