# Supplementary material for: Conservation of the Hydrogen-Bonded Pyridone Homosynthon in Halogen-Bonded Cocrystals
Source: Cryst Growth Des. 2022 Jan 10;22(2):987–92. doi: 10.1021/acs.cgd.1c01424 (PMC8861932; doi:10.1021/acs.cgd.1c01424)
Supplement: Supplementary file 1 — cg1c01424_si_001.pdf [file cg1c01424_si_001.pdf]

Conservation of the Hydrogen-bonded Pyridone Homosynthon in Halogen-bonded Cocrystals

Nikola Bedeković,[a] Luka Fotović,[a] Vladimir Stilinović,[a]* Dominik Cinčić[a]*

[a]University of Zagreb, Faculty of Science, Department of Chemistry, Horvatovac 102A, 10 000 Zagreb, Croatia; nbedekovic@chem.pmf.hr (N.B.), lfotovic@chem.pmf.hr (L.F.) vstilinovic@chem.pmf.hr (V.S.), dominik@chem.pmf.hr (D.C.)

* Correspondence: vstilinovic@chem.pmf.hr (V.S.); Tel.: +385-1-4606-371 (V.S.); dominik@chem.pmf.hr (D.C.); Tel.: +385-1-4606-362 (D.C.)

Table of Content

Item	Page
**Figures S1–S7** (ORTEP representations of the formula units of the prepared compounds)	1
**Figures S8** (PXRD patterns of the prepared compounds)	3
**Figures S9–S15** (DSC and TG curves of the prepared compounds)	3
**Figure S16–S23 (**IR spectra of the prepared compounds)	7
**Table S1** Crystallographic data	11
**Table S2.** Halogen bond lengths and their relative shortenings (rs) in obtained compounds.	14
Preparation of the cocrystals	15
X-Ray Diffraction Measurements	15
Thermal analysis	15
References	16

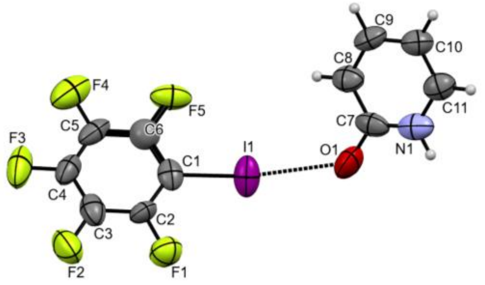

Figure S1. Molecular structure of **b3pm** showing the atom-labelling scheme. Displacement ellipsoids are drawn at the 50 % probability level, and H atoms are shown as small spheres of arbitrary radius.

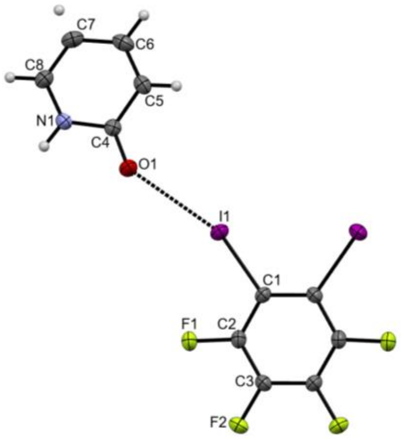

Figure S2. Molecular structure of (**b4pm**)(**13tfib**) showing the atom-labelling scheme. Displacement ellipsoids are drawn at the 50 % probability level, and H atoms are shown as small spheres of arbitrary radius.

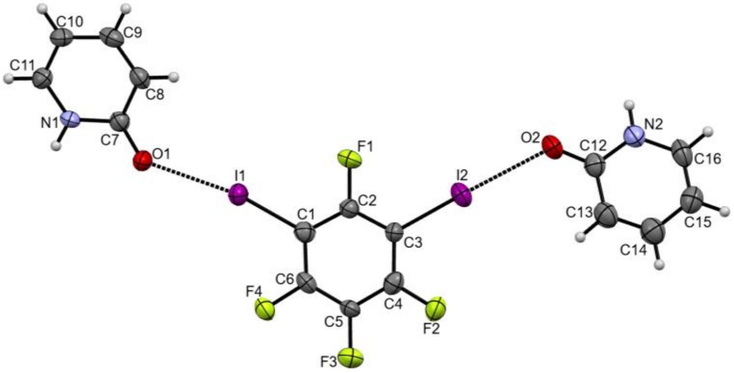

Figure S3. Molecular structure of **(b4pm)(13tfib)** showing the atom-labelling scheme. Displacement ellipsoids are drawn at the 50 % probability level, and H atoms are shown as small spheres of arbitrary radius.

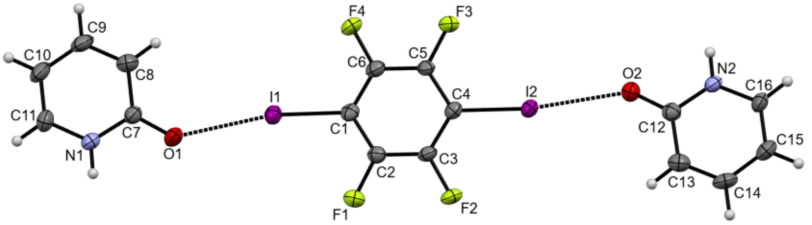

Figure S4. Molecular structure of **(b4pm)$_2$(14tfib)** showing the atom-labelling scheme. Displacement ellipsoids are drawn at the 50 % probability level, and H atoms are shown as small spheres of arbitrary radius.

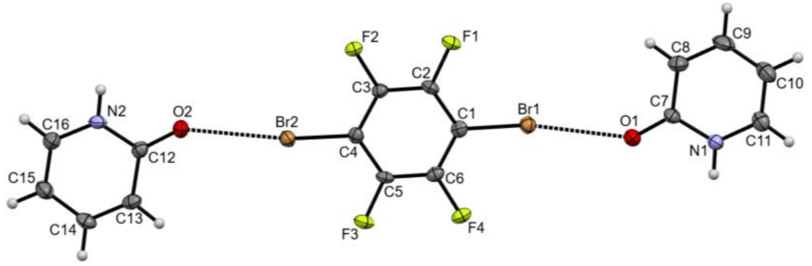

Figure S5. Molecular structure of (**b4pm**)(**ipfb**) showing the atom-labelling scheme. Displacement ellipsoids are drawn at the 50 % probability level, and H atoms are shown as small spheres of arbitrary radius.

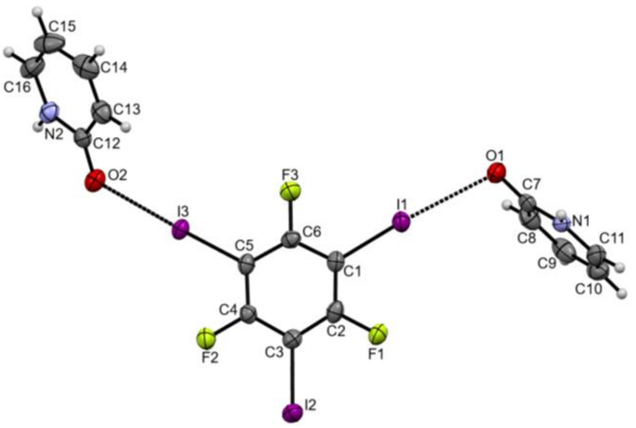

Figure S6. Molecular structure of (**b4pm**)(**135tfib**) showing the atom-labelling scheme. Displacement ellipsoids are drawn at the 50 % probability level, and H atoms are shown as small spheres of arbitrary radius.

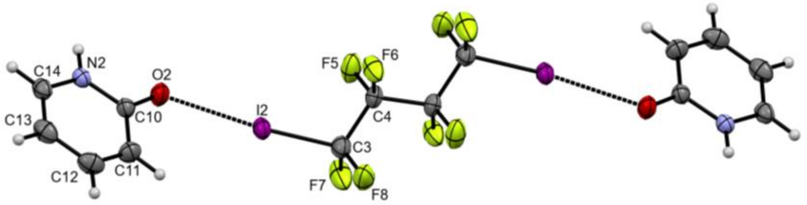

Figure S7. Molecular structure of (**b4pm**)(**135tfib**) showing the atom-labelling scheme. Displacement ellipsoids are drawn at the 50 % probability level, and H atoms are shown as small spheres of arbitrary radius.

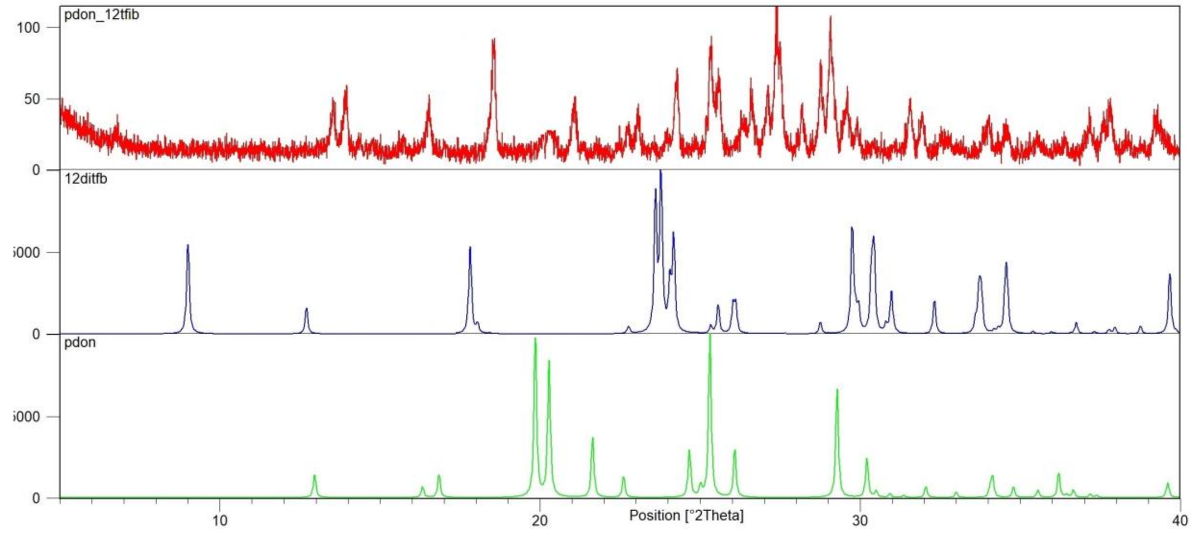

Figure S8. Comparison of the PXRD patterns of (**12ditfb**)(**pdon**)$_2$, **12ditfb** and **pdon**.

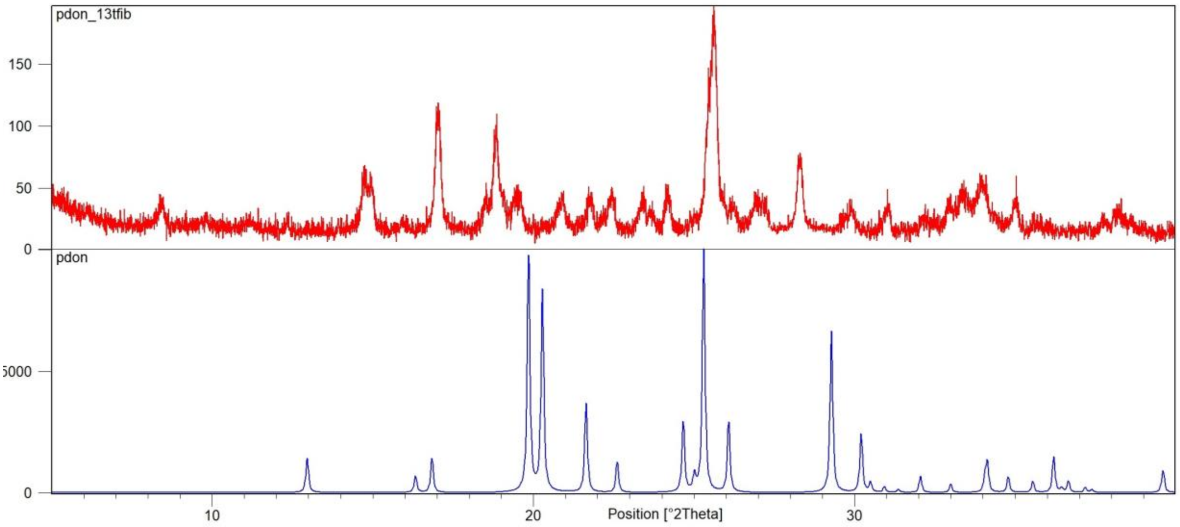

Figure S9. Comparison of the PXRD patterns of (**13ditfb**)(**pdon**)$_2$ and **pdon**.

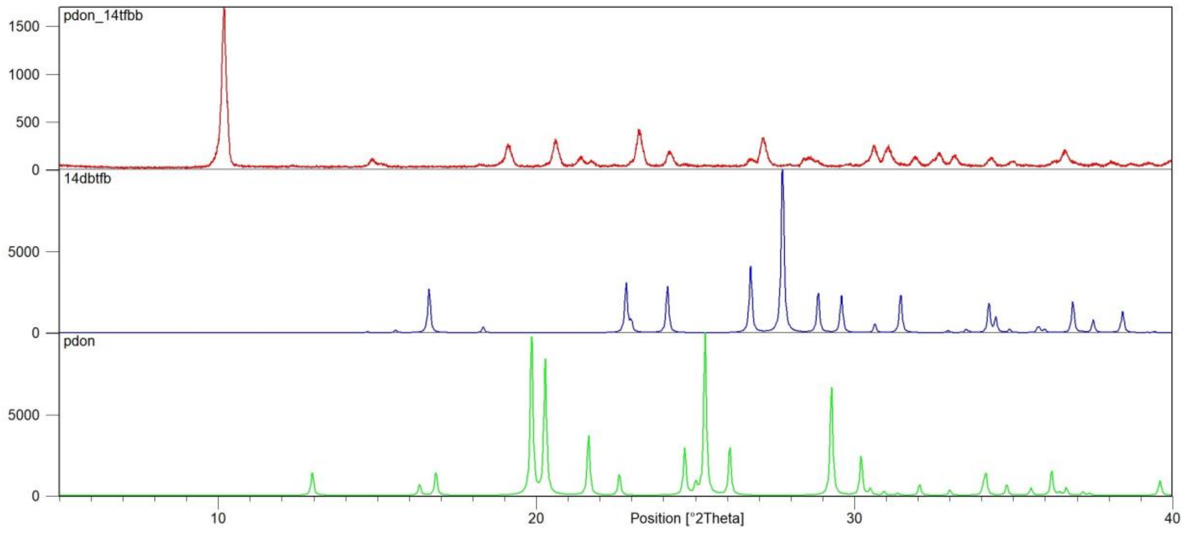

Figure S10. Comparison of the PXRD patterns of (**14dbtfb**)(**pdon**)$_2$, **14dbtfb** and **pdon**.

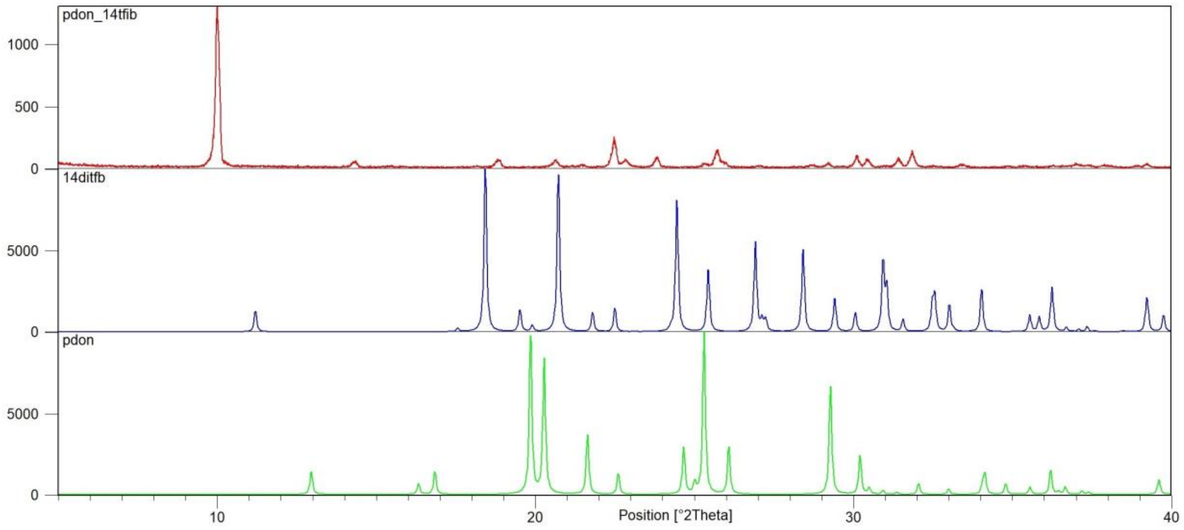

Figure S11. Comparison of the PXRD patterns of (**14ditfb**)(**pdon**)$_2$, **14ditfb** and **pdon**.

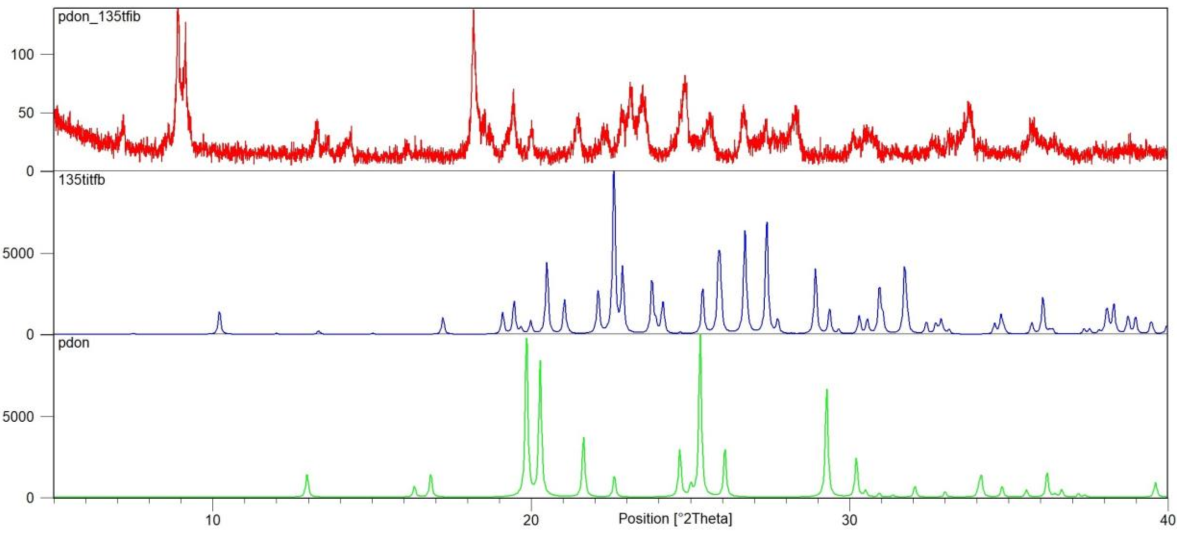

Figure S12. Comparison of the PXRD patterns of (**135titfb**)(**pdon**)$_2$, **135titfb** and **pdon**.

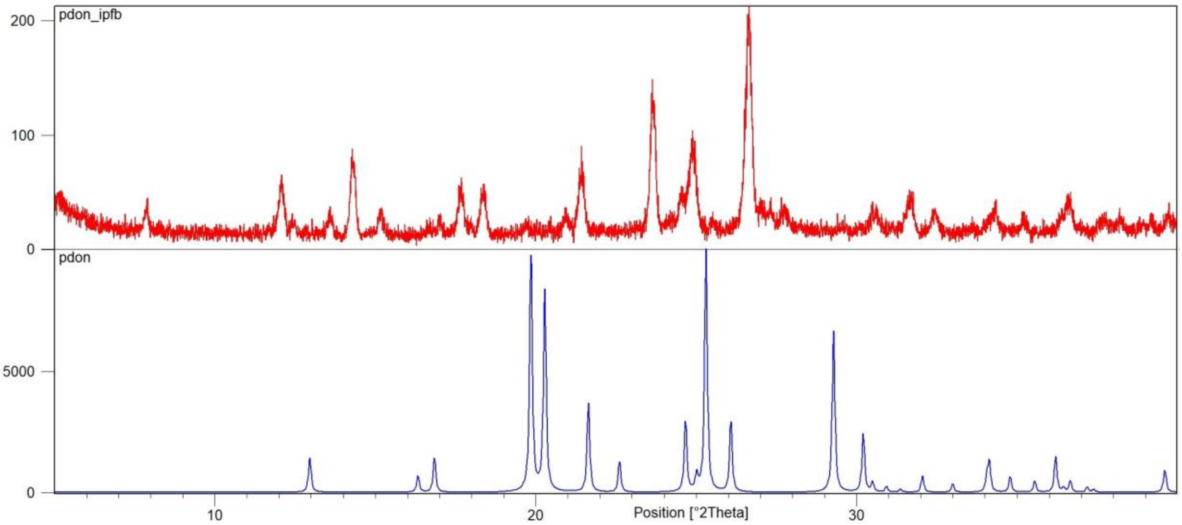

Figure S13. Comparison of the PXRD patterns of (**ipfb**)(**pdon**) and **pdon**.

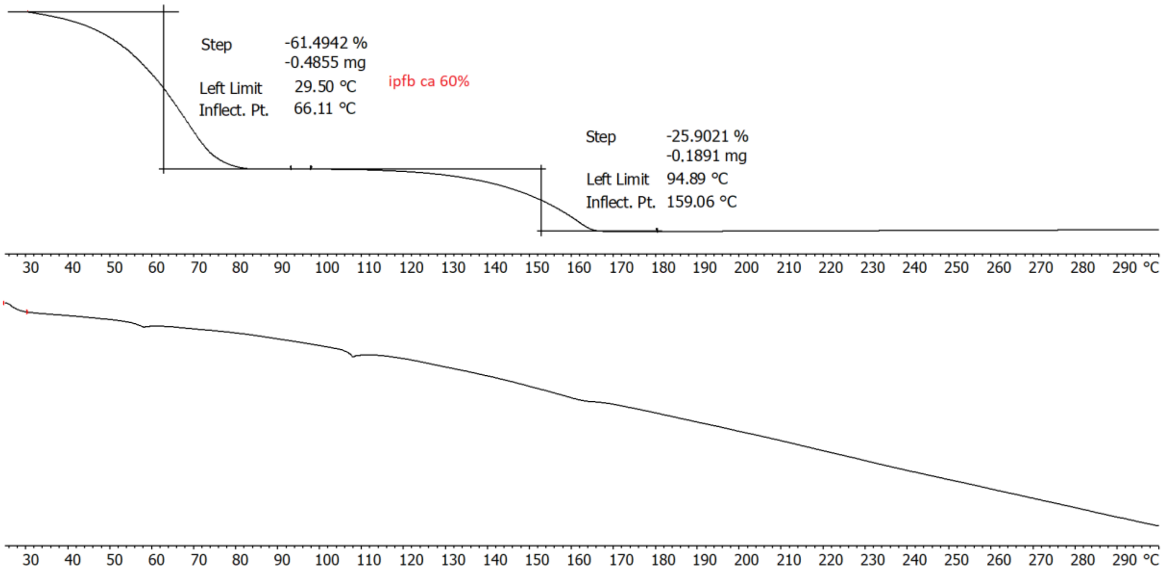

Figure S14. TG and DSC curves of the (**ipfb**)(**pdon**).

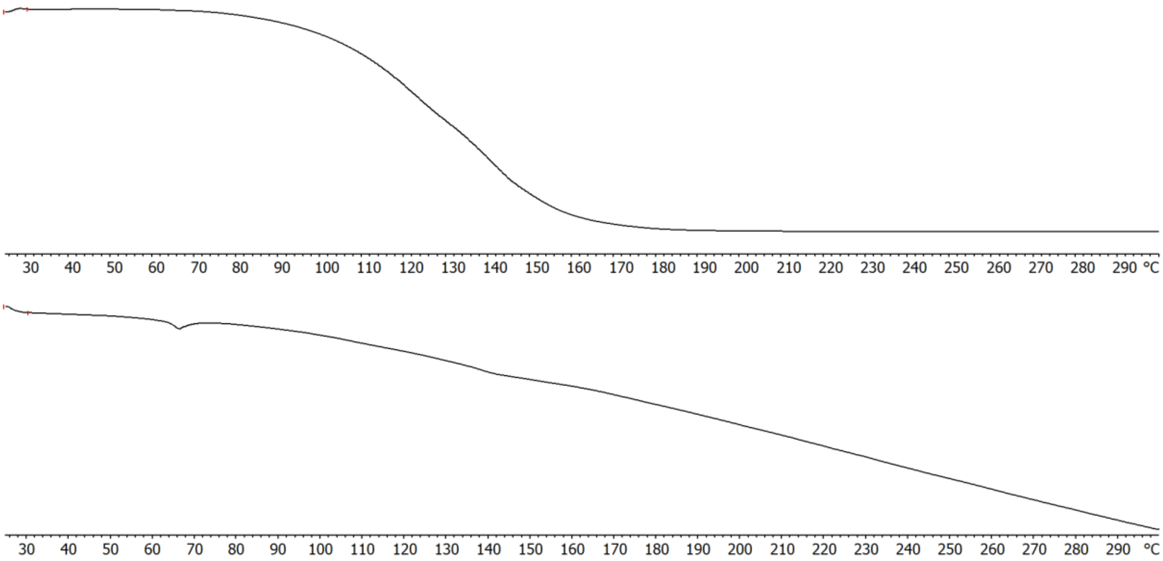

Figure S15. TG and DSC curves of the (**12ditfb**)(**pdon**)$_2$.

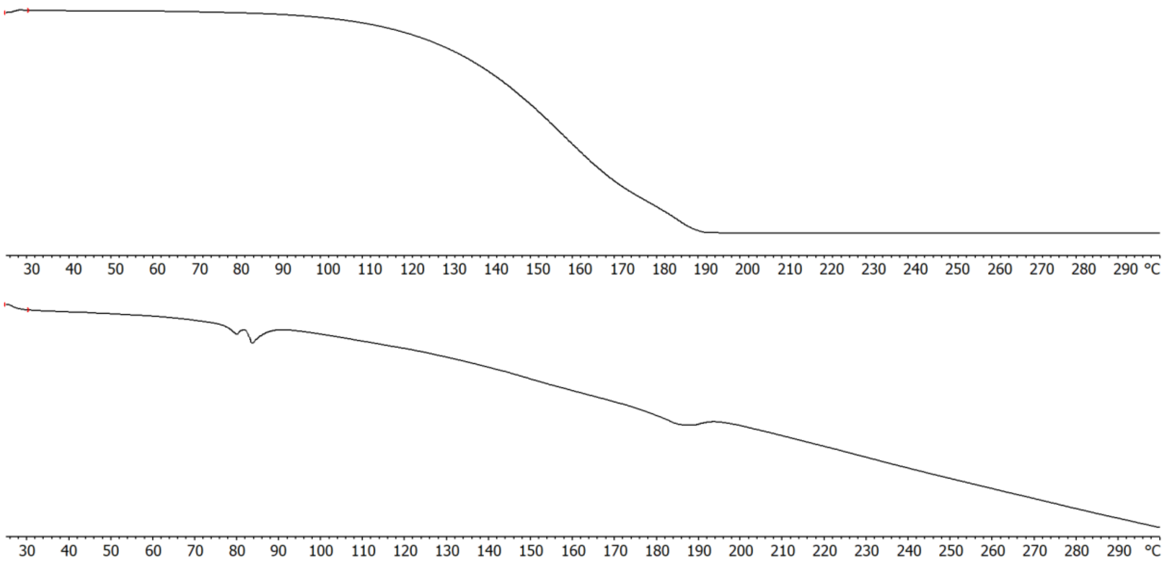

Figure S16. TG and DSC curves of the (**13ditfb**)(**pdon**)$_2$.

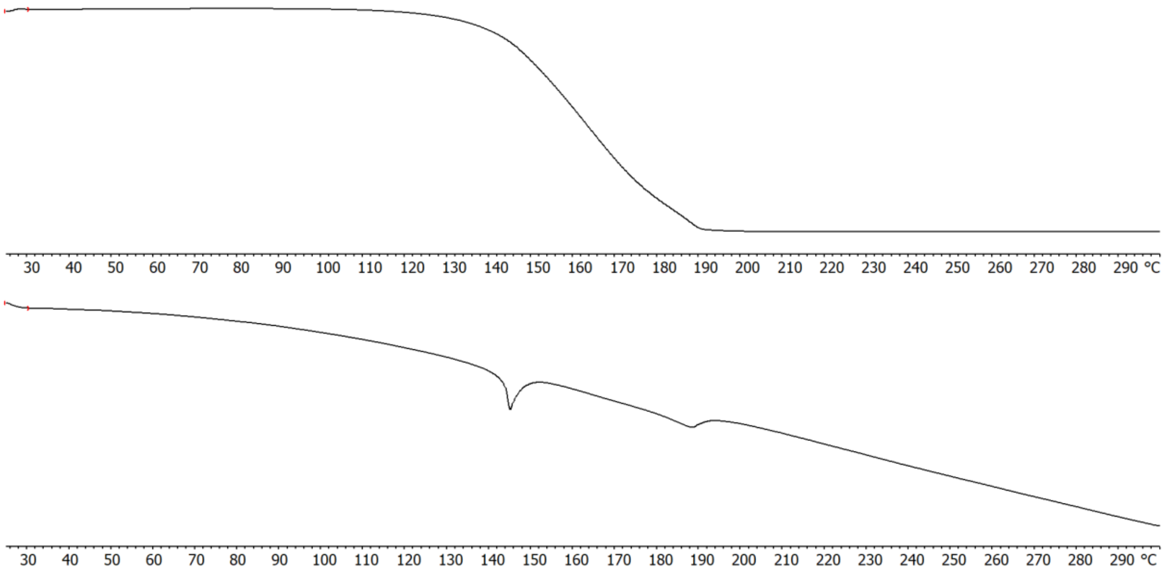

Figure S17. TG and DSC curves of the (**14ditfb**)(**pdon**)$_2$.

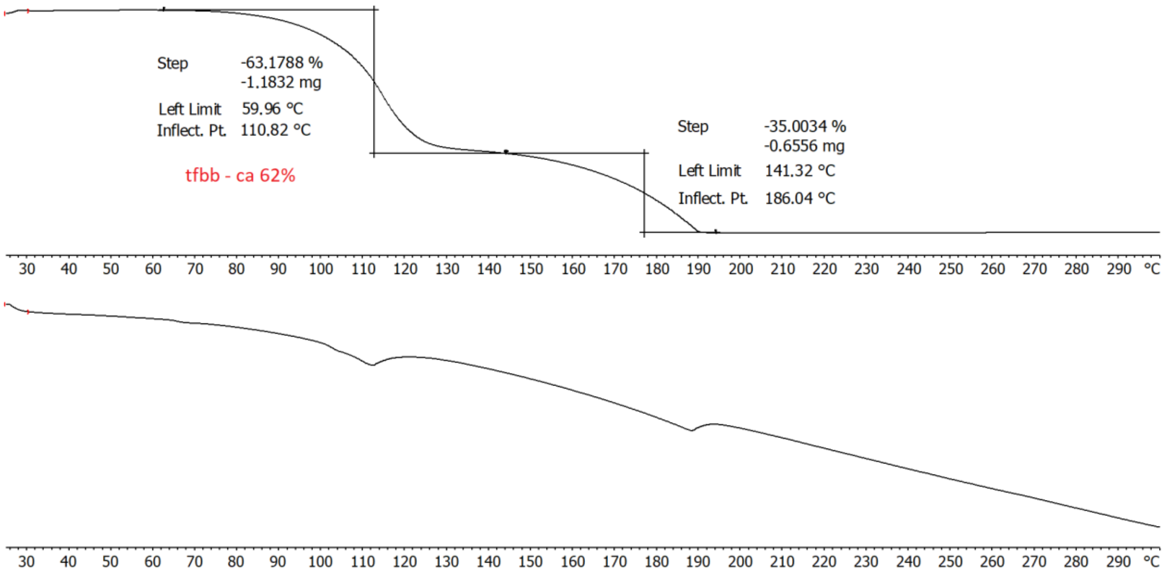

Figure S18. TG and DSC curves of the (**13dbtfb**)(**pdon**)2.

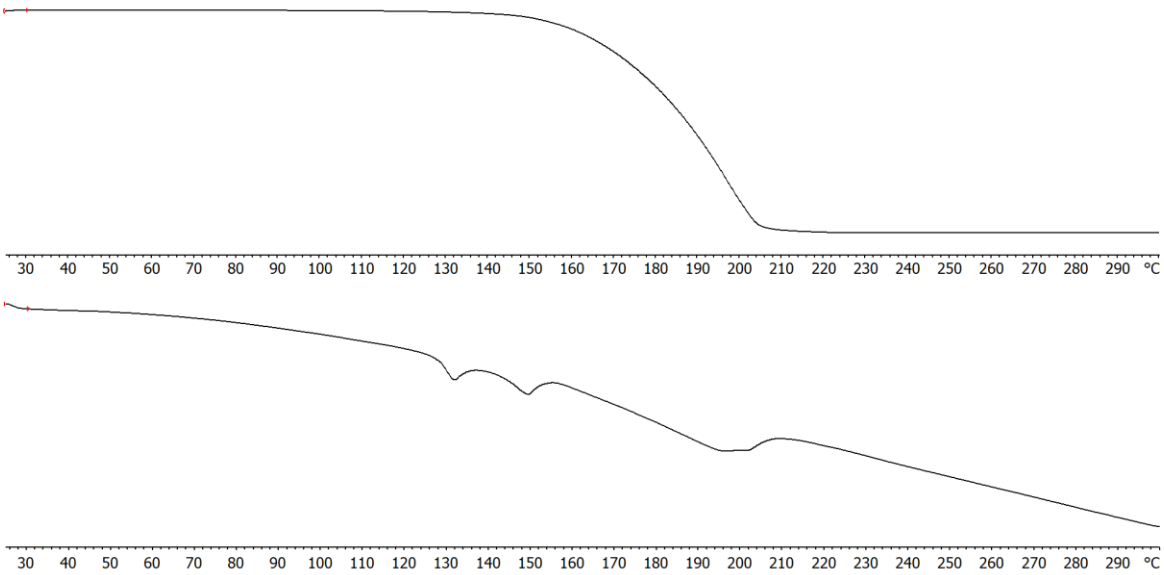

Figure S19. TG and DSC curves of the (**135titfb**)(**pdon**)2.

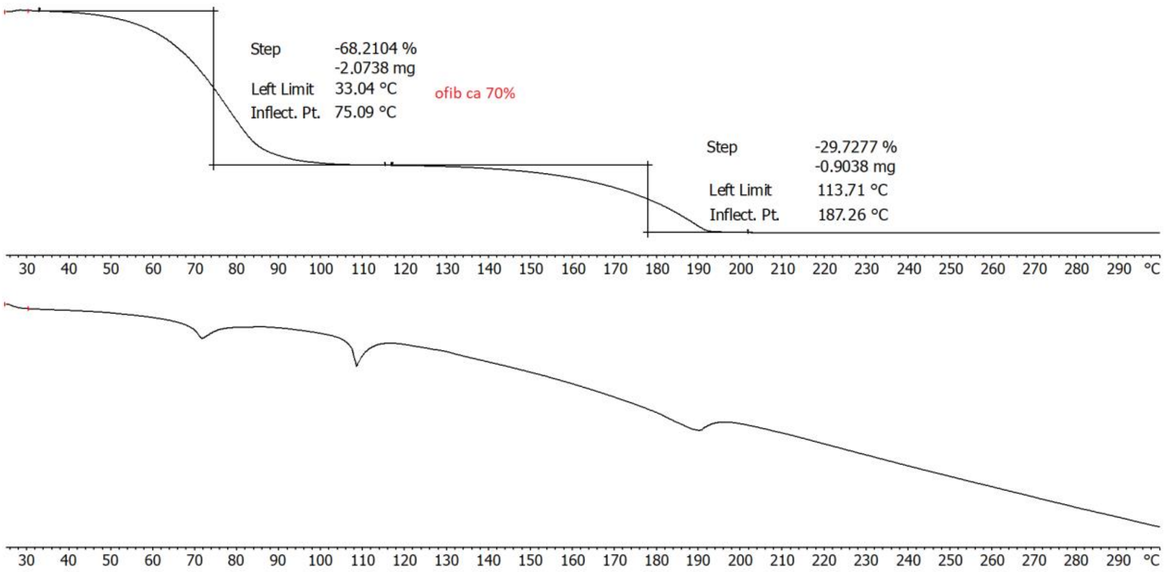

Figure S20. TG and DSC curves of the (**ofib**)(**pdon**)$_2$.

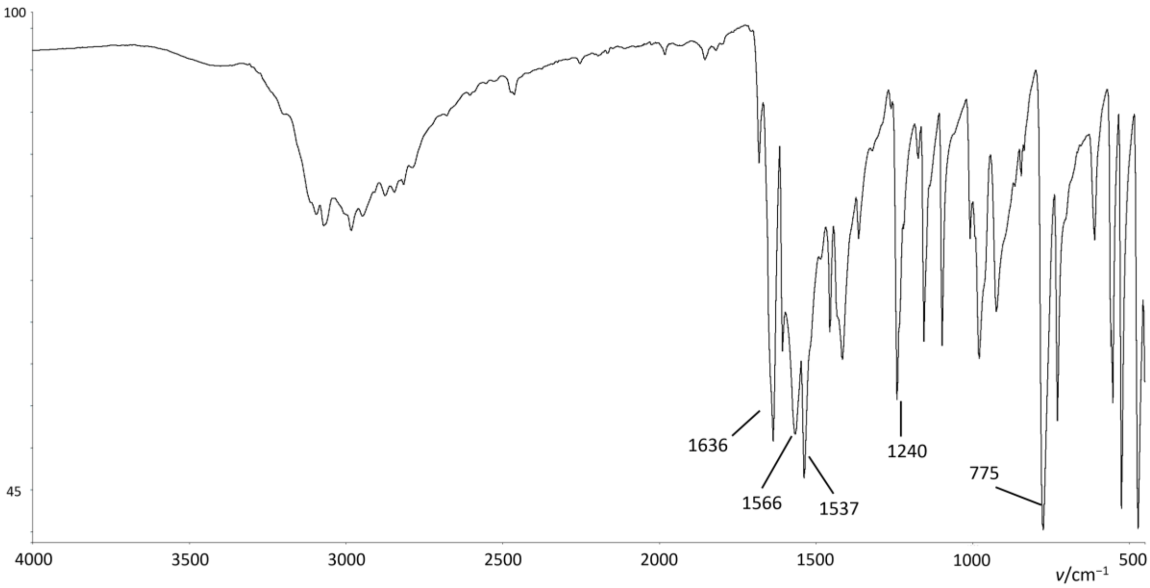

Figure S21. IR spectrum of the **pdon**.

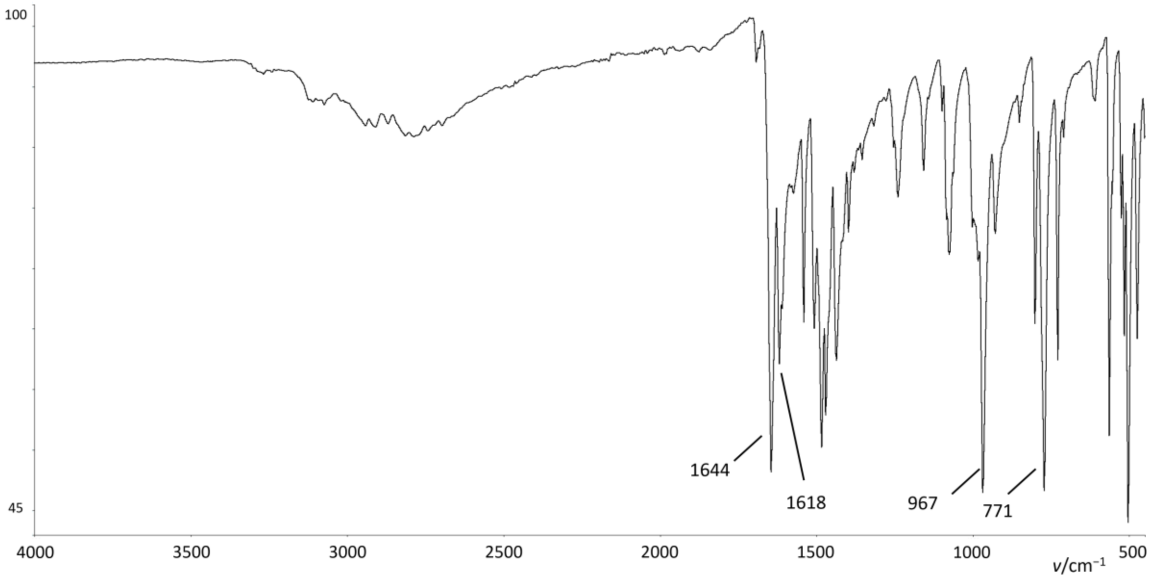

Figure S22. IR spectrum of the (**ipfb**)(**pdon)**.

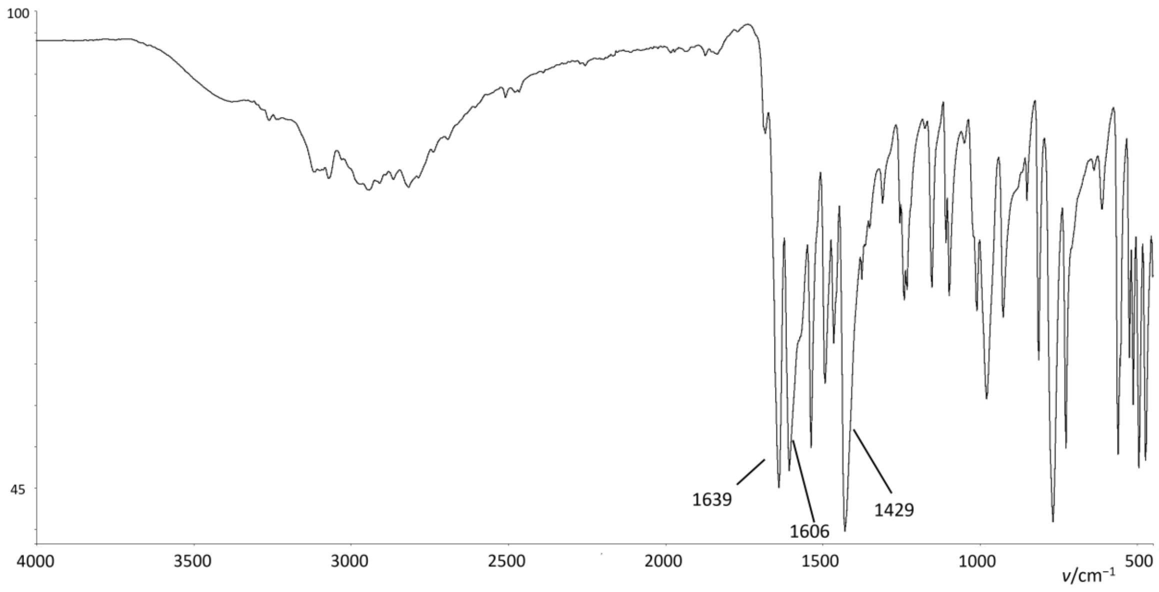

Figure S23. IR spectrum of the (**12ditfb**)(**pdon)**$_2$.

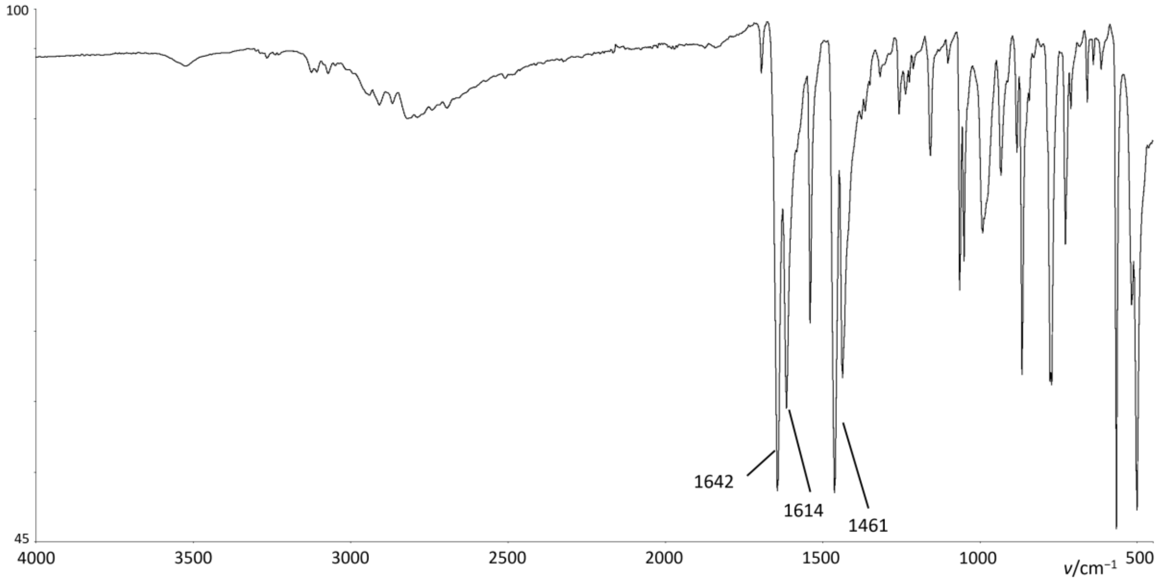

Figure S24. IR spectrum of the (**13ditfb**)(**pdon)**$_2$.

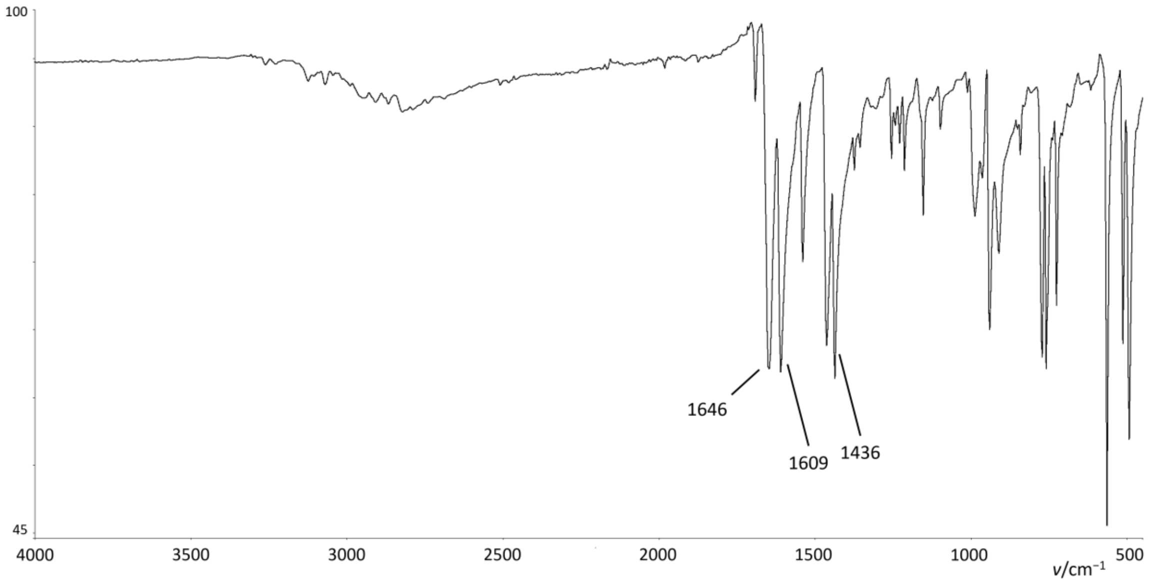

Figure S25. IR spectrum of the (**14ditfb**)(**pdon)**2.

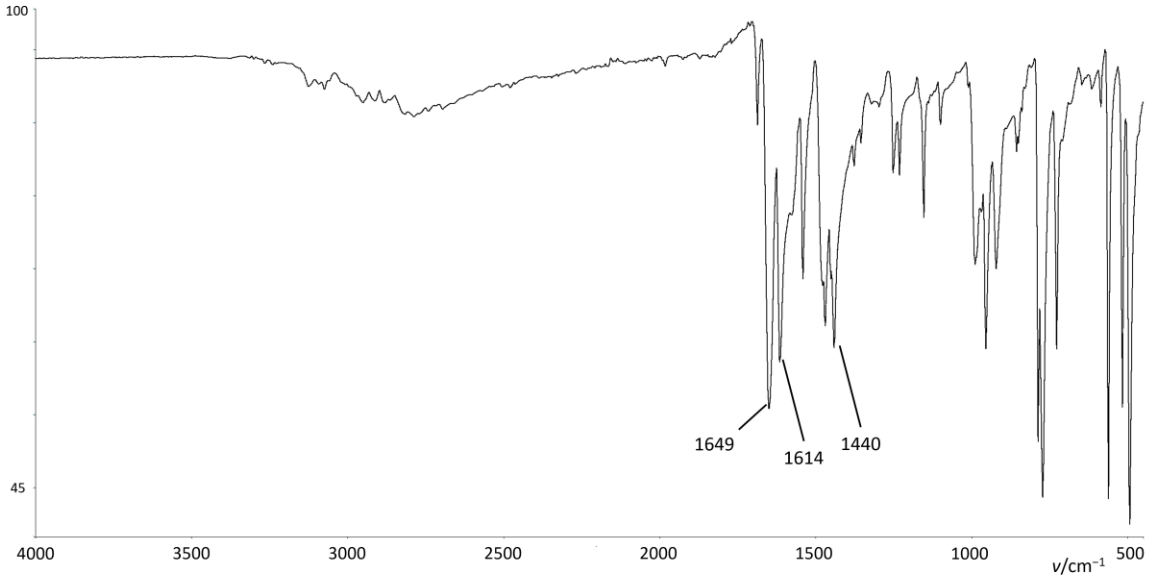

Figure S26. IR spectrum of the (**14dbtfb**)(**pdon)**2.

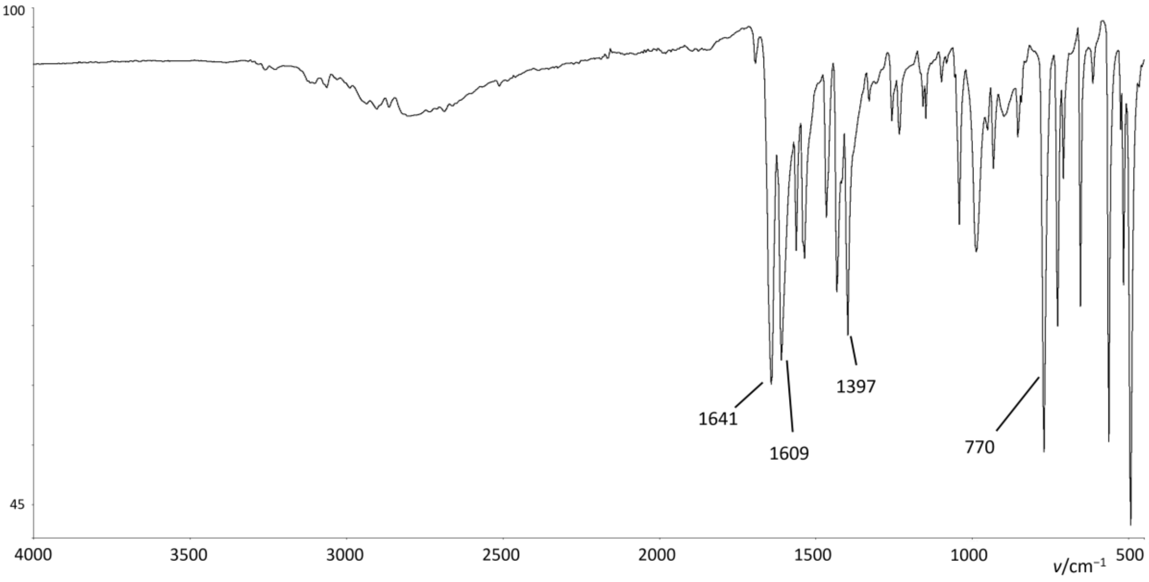

Figure S27. IR spectrum of the (**135titfb**)(**pdon)**$_2$.

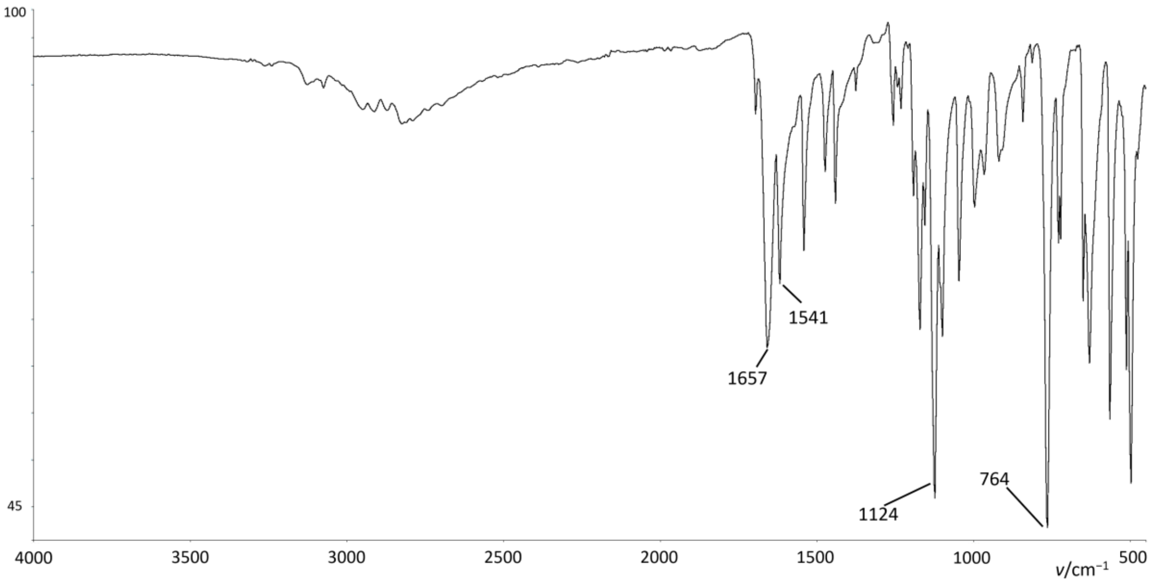

Figure S28. IR spectrum of the (**ofib**)(**pdon)**$_2$.

Table S1. An overview and crystallographic data of the prepared compounds.

	**(ipfb)(pdon)**	(**12ditfb**)(**pdon**)$_2$	(**13ditfb**)(**pdon**)$_2$
Molecular formula	$C_{11}H_5NOF_5I$	$C_{16}H_{10}N_2O_2I_2F_4$	$C_{16}H_{10}N_2O_2I_2F_4$
M_r	389.07	592.07	592.07
Crystal system	monoclinic	monoclinic	monoclinic
Space group	$P2_1/c$	$I2/a$	$P2_1/c$
a / Å	7.4107(2)	13.8467(5)	10.8798(6)
b / Å	7.4217(1)	11.4250(2)	10.3443(4)
c / Å	22.9779(5)	12.8921(5)	16.8469(8)
α / °	90	90	90
β / °	96.657(2)	118.083(4)	100.921(5)
γ / °	90	90	90
V / Å^3	1255.26(3)	1799.39(44)	1861.68(18)
Z	4	4	4
ρ_{calc} / g cm^{-3}	2.03	2.19	2.11
T / K	170	170	170
μ / mm^{-1}	2.602	3.548	3.429
F(000)	716.0	1112.0	1112.0
Refl. collected/unique	32945/2743	14097 / 1972	31243 / 4063
parameters	172	118	235
$\Delta\rho_{max}$, $\Delta\rho_{min}$ / e Å^{-3}	5.882; –5.596	0.530; –1.646	3.300; –1.139
$R[F^2 > 4\sigma(F^2)]$	0.141	0.029	0.069
w$R(F^2)$	0.253	0.073	0.150
Goodness of fit, S	1.080	1.168	1.052

Table A1. Continuation

Table S1. Continuation

	**(14ditfb)(pdon)**$_2$	**(14dbtfb)(pdon)**$_2$	**(135titfb)(pdon)**$_2$
Molecular formula	$C_{16}H_{10}N_2O_2I_2F_4$	$C_{16}H_{10}N_2O_2Br_2F_4$	$C_{16}H_{10}N_2O_2I_3F_3$
M_r	592.07	498.07	699.97
Crystal system	monoclinic	monoclinic	triclinic
Space group	$P2_1/c$	$P2_1/n$	$P{-1}$
a / Å	12.5097(3)	12.4129(5)	7.7093(2)
b / Å	8.1933(2)	7.8380(3)	10.8881(3)
c / Å	18.6002(4)	18.2390(8)	13.4276(4)
α / °	90	90	68.054(2)
β / °	106.459(3)	107.876(5)	84.115(2)
γ / °	90	90	77.422(2)
V / Å^3	1828.32(17)	1688.85(29)	1020.07(10)
Z	4	8	2
ρ_{calc} / g cm^{-3}	2.15	1.96	2.28
T / K	170	170	170
μ / mm^{-1}	3.492	4.855	4.635
F(000)	1112.0	968.0	644.0
Refl. collected/unique	29682 / 3985	14483 / 3305	16558 / 4448
parameters	235	235	235
$\Delta\rho_{max}$, $\Delta\rho_{min}$ / e Å^{-3}	0.883; –2.068	1.200; –1.882	1.319; –1.090
$R[F^2 > 4\sigma(F^2)]$	0.042	0.062	0.046
w$R(F^2)$	0.112	0.171	0.118
Goodness of fit, S	1.155	1.053	1.043

Table S1. Continuation

	(**ofib**)(**pdon**)$_2$
Molecular formula	$C_{14}H_{10}N_2O_2I_2F_8$
M_r	643.88
Crystal system	monoclinic
Space group	$C2/c$
a / Å	27.4114(6)
b / Å	7.0340(2)
c / Å	20.2776(5)
α / °	90
β / °	98.227(2)
γ / °	90
V / Å^3	3869.52(11)
Z	8
ρ_{calc} / g cm^{-3}	2.21
T / K	170
μ / mm^{-1}	3.338
F(000)	2416.0
Refl. collected/unique	35205 / 5641
parameters	253
$\Delta\rho_{max}$, $\Delta\rho_{min}$ / e Å^{-3}	1.206; –0.746
$R[F^2 > 4\sigma(F^2)]$	0.034
w$R(F^2)$	0.069
Goodness of fit, S	1.072

Table S2. Halogen bond lengths and their relative shortenings (rs) in obtained compounds.

compound	*d*(I···O) / Å	*d*-r_{vdw} / Å	*rs* / %
(**12ditfb**)(**pdon**)$_2$	3.041	-0.459	13.1
(**13ditfb**)(**pdon**)$_2$	2.765	-0.735	21.0
	2.817	-0.683	19.5
(**14ditfb**)(**pdon**)$_2$	2.795	-0.705	20.1
	2.807	-0.693	19.8
(**135ditfb**)(**pdon**)$_2$	2.840	-0.660	18.9
	2.920	-0.580	16.6
	2.941	-0.559	16.0
(**14dbtfb**)(**pdon**)$_2$	2.876	-0.494	14.7
	2.925	-0.445	13.2
(**ipfb**)(**pdon**)	2.698	-0.802	22.9
(**ofib**)(**pdon**)$_2$	2.775	-0.725	20.7
	2.789	-0.711	20.3

Preparation of the cocrystals

Cocrystals were prepared by dissolving **pdon** (10 mg, 105.1 mmol) and corresponding halogen bond donor (Table 2) in 3 mL of hot acetone/ethanol mixture (1:1). Single crystals suitable for X-ray structure determination were obtained after cooling to the room temperature (ca. 25 °C).

Table S2. Masses and volumes of the halogen bond donors.

**XB donor**	mass / volume
ipfb	16 μL
12ditfb	89 mg
13ditfb	31 μL
14ditfb	89 mg
14dbtfb	68 mg
135titfb	166 mg
ofib	41 μL

X-Ray Diffraction Measurements

All single crystal X-ray diffraction experiments were performed using an Oxford Diffraction XtaLAB Synergy, Dualflex, HyPix Xray four-circle diffractometer with mirror-monochromated Mo*Kα* (*λ* = 0.71073 Å) radiation. The data sets were collected using the ω -scan mode over the 2 θ -range up to 60°. Programs CrysAlis PRO CCD and CrysAlis PRO RED were employed for data collection, cell refinement, and data reduction.[1, 2] The structures were solved by SHELXT or by direct methods using the SHELXS and refined using SHELXL programs.[3, 4] The structural refinement was performed on F2 using all data. The hydrogen atoms were placed in calculated positions and treated as riding on their parent atoms [C–H = 0.93 Å and Uiso(H) = 1.2 Ueq(C); C–H = 0.97 Å and Uiso(H) = 1.2 Ueq(C)]. All calculations were performed using the WinGX or Olex2 1.3-ac4 crystallographic suite of programs.[5] Further details are available from the Cambridge Crystallographic Centre. Molecular structures of compounds and their packing diagrams were prepared using Mercury.[6]

Thermal analysis

Differential scanning calorimetry (DSC) and thermogravimetric (TG) measurements were performed simultaneously on a Mettler-Toledo TGA/DSC 3+ module (Mettler Toledo, Greifensee, Switzerland). Samples were placed in alumina crucibles (40 μL)

and heated 25 to 300 °C, at a heating rate of 10 °C min-1 under nitrogen flow of 150 mL min-1.

Data collection and analysis were performed using the program package STARe Software (Version 15.00, Mettler Toledo, Greifensee, Switzerland).[7] TG and DSC thermograms of the prepared compounds are shown in Figures S11–S15 in Supplementary Materials.

References:

(1) Manual, U. CrysAlis Pro. Power. Agilent Technologies Ltd: Yarnton, Oxfordshire, England 2014.

(2) CrysAlis PRO CCD. Agilent Technologies Ltd: Yarnton, Oxfordshire, England 2014.

(3) Sheldrick, G. M. Crystal Structure Refinement with SHELXL. Acta Crystallogr. Sect. C Struct. Chem. 2015, 71 (1). https://doi.org/10.1107/S2053229614024218.

(4) Sheldrick, G. M. SHELXT – Integrated Space-Group and Crystal-Structure Determination. Acta Crystallogr. Sect. A Found. Adv. 2015, 71 (1). https://doi.org/10.1107/S2053273314026370.

(5) Farrugia, L. J. WinGX Suite for Small-Molecule Single-Crystal Crystallography. J. Appl. Crystallogr. 1999, 32 (4). https://doi.org/10.1107/S0021889899006020.

(6) Macrae, C. F.; Bruno, I. J.; Chisholm, J. A.; Edgington, P. R.; McCabe, P.; Pidcock, E.; Rodriguez-Monge, L.; Taylor, R.; van de Streek, J.; Wood, P. A. Mercury CSD 2.0 – New Features for the Visualization and Investigation of Crystal Structures. J. Appl. Crystallogr. 2008, 41 (2). https://doi.org/10.1107/S0021889807067908.

(7) STARe Software. Mettler Toledo: Greifensee, Switzerland 2016.
